# Supplementary material for: Concordance of CSF measures of Alzheimer's pathology with amyloid PET status in a preclinical cohort: A comparison of Lumipulse and established immunoassays
Source: Alzheimers Dement (Amst). 2020 Sep 13;12(1):e12097. doi: 10.1002/dad2.12097 (PMC7503103; doi:10.1002/dad2.12097)
Supplement: Supplementary file 1 — Supporting information. [file DAD2-12-e12097-s001.docx]

**Supplementary online content**

Supplementary Methods

*A40 interference*


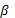

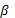


To test for the effect of A40 interference on measurement of A42, recombinant A40 (Ultrapure, TFA, rPeptide, Bogart) was spiked into two CSF samples with known low and high concentrations of native A42. The “low CSF A42” sample had an A42 concentration of 240 pg/ml and the “high CSF A42” sample had an A42 concentration of 950 pg/ml as quantified using the INNOTEST® -amyloid 1-42 (Fujirebio) assay. Samples underwent two freeze-thaw cycles before being used for this experiment. Spiking solutions were made by suspending the A40 peptide in 1% NH_4_OH and sonicating before diluting with the diluent provided in each assay kit. To minimize differences in matrix effects, the volume of spiking solution was fixed as 10% of the volume of the final sample. Final spiking concentrations of A40 were 10, 20, 30, 40, 50 and 60 ng/ml. Each spiked sample was assayed in duplicate on each of the three platforms to measure A42 (Lumipulse, MSD and INNOTEST).


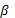

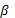

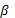

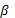

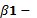

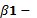

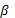

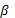

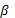

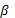

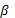

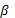

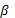

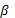

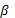

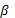

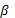

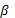

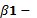

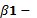

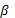

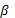

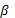

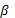


| Platform | INNOTEST | | | MSD | |
| --- | --- | --- | --- | --- | --- |
| Biomarker | A42 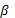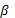 | t-tau | p-tau181 | A40 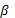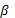 | A42 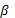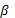 |
| Low run validation control intra-run CV %  Plate 1  Plate 2 | 2.5  10.3 | 3.9  27.8 | 0.2  1.6 | 5.4  0.2 | 2.3  1.9 |
| High run validation control intra-run CV %  Plate 1  Plate 2 | 2.2  6.5 | 1.0  9.0 | 9.2  1.8 | 5.4  5.7 | 7.7  4.8 |
| Low CSF control inter-run CV % | 15.1 | 17.9 | 7.7 | 3.1 | 8.1 |
| High CSF control inter-run CV % | 7.4 | 5.2 | 7.9 | 3.9 | 4.7 |
| Sample CV range % (n) | 0.08 - 26.3  (72) | 0 – 14.6  (70) | 0.01 – 26.8  (72) | 0.01 – 24.3  (72) | 0.04 – 22.9  (71) |

Supplementary table 1: Quantification variation for the INNOTEST and MSD assays. The sample CV range was capped at 30% and n reflects those remaining after exclusion of samples that had a CV above this.

Abbreviations: CV, coefficient of variation

Supplementary table 2: Comparison of characteristics of individuals included vs excluded from this study

|  | All included in PET analysis  n=63 unless otherwise stated | Individuals excluded due to missing PET or CSF data  n=9 unless otherwise stated | *P* | All individuals with available data | |
| --- | --- | --- | --- | --- | --- |
|  |  |  |  |  | n |
| *Demographics* |  |  |  |  |  |
| Mean age at CSF sampling (SD), years | 72.7 (1.3) | 72.3 (0.2) | 0.357 | 72.6 (1.2) | 72 |
| Sex, % male | 71.4 | 55.6 | 0.334 | 69.4 | 72 |
| *APOE* 4 carrier status, % carrying one or two alleles 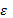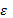 | 22.6, n=62 | 44.4 | 0.159 | 25.4 | 71 |
| Median MMSE (IQR) | 29 (28, 30) | 30 (28, 30) | 0.260 | 29 (28, 30) | 72 |
| *Lumipulse platform results* |  |  |  |  |  |
| Median CSF A40 (IQR), pg/ml 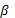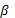 | 13193 (10528, 16376) | 12288 (10744, 14025) | 0.568 | 12975 (10586, 16246) | 72 |
| Median CSF A42 (IQR), pg/ml 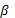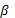 | 1654 (1181, 2338) | 1490 (1017, 1853), n=8 | 0.267 | 1639 (1139, 2206) | 71 |
| Median CSF A42/A40 (IQR) ratio 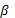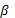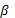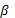 | 0.145 (0.105, 0.156) | 0.132 (0.082, 0.152), n=8 | 0.344 | 0.142 (0.098, 0.159) | 71 |
| Median CSF t-tau (IQR) , pg/ml | 356 (311, 444) | 318 (270, 415) | 0.223 | 349 (310, 444) | 72 |
| Median CSF p-tau181 (IQR), pg/ml | 47.5 (36.8, 57.8) | 46.2 (30.9, 67.9) | 0.973 | 47.1 (36.0, 57.9) | 72 |
| Median CSF A42/t-tau ratio (IQR) 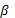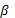 | 4.81 (3.07, 6.44) | 4.42 (2.87, 8.19), n=8 | 0.957 | 4.81 (2.98, 6.44) | 71 |
| Median CSF A42/p-tau181 ratio (IQR) 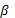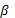 | 42.0 (21.5, 50.4) | 39.2 (18.1, 54.4), n=8 | 0.716 | 41.3 (20.9, 50.4) | 71 |
| *Mesoscale Discovery Platform results* |  |  |  |  |  |
| Median CSF A38 (IQR), pg/ml 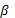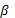 | 3171 (2554, 3778) | 3236 (2514, 3482) | 0.845 | 3179 (2562, 3694) | 72 |
| Median CSF A40 (IQR), pg/ml 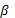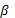 | 7066 (6254, 8338) | 7076 (5674, 7417) | 0.628 | 7071 (6264, 8309) | 72 |
| Median CSF A42 (IQR), pg/ml 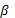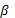 | 739 (514, 857) | 803 (470, 873), n-8 | 0.771 | 750 (54, 857) | 71 |
| Median CSF A42/A40 (IQR) ratio 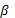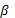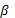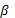 | 0.108 (0.081, 0.117) | 0.115 (0.082, 0.124), n=8 | 0.383 | 0.110 (0.081, 0.118) | 71 |
| *INNOTEST platform results* |  |  |  |  |  |
| Median CSF A42 (IQR), pg/ml 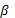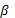 | 1111 (815, 1406) | 1090 (811, 1317) | 0.832 | 1100 (815, 1399) | 72 |
| Median CSF t-tau (IQR), pg/ml | 372 (277, 436) | 327 (217, 378), n=7 | 0.384 | 366 (273, 436) | 70 |
| Median CSF p-tau181 (IQR), pg/ml | 57.3 (43.3, 70.8) | 54.0 (41.3, 71.8) | 0.953 | 56.5 (43.7, 70.6) | 72 |
| Median CSF A42/t-tau ratio (IQR) 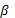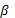 | 3.56 (2.32, 4.57) | 4.15 (1.66, 5.49), n=7 | 0.762 | 3.58 (2.16, 4.59) | 70 |
| Median CSF p-tau181/A42 ratio (IQR) 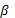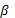 | 21.4 (15.2, 27.6) | 20.8 (12.4, 27.5) | 0.832 | 21.3 (15.4, 27.5) | 72 |

Supplementary table 3: Concordance between CSF and PET biomarkers by incorporation of age, sex and APOE ε4 carrier status into predictive models (n = 62)

|  |  | **Biomarker alone** | | **Biomarker + Age + Sex + *APOE* 4 carrier status** 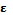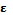 | |
| --- | --- | --- | --- | --- | --- |
| **Biomarker** | **Platform** | **AUC** | **95% CI for AUC** | **AUC** | **95% CI for AUC** |
| **None** | - | - | - | 0.776 | 0.634 – 0.917 |
| **Aβ42** | Lumipulse | 0.889 | 0.807 – 0.970 | 0.920 | 0.850 – 0.990 |
|  | MSD | 0.895 | 0.817 – 0.973 | 0.929 | 0.868 – 0.991 |
|  | INNOTEST | 0.947 | 0.893 – 1.000 | 0.962 | 0.918 – 1.000 |
| **t-tau** | Lumipulse | 0.667 | 0.483 – 0.852 | 0.846 | 0.719 – 0.973 |
|  | INNOTEST | 0.831 | 0.716 – 0.945 | 0.900 | 0.818 – 0.981 |
| **p-tau181** | Lumipulse | 0.885 | 0.797 – 0.974 | 0.926 | 0.855 – 0.998 |
|  | INNOTEST | 0.804 | 0.668 – 0.939 | 0.887 | 0.787 – 0.987 |
| **Aβ42/Aβ40** | Lumipulse | 0.966 | 0.919 – 1.000 | 0.980 | 0.946 – 1.000 |
|  | MSD | 0.966 | 0.910 – 1.000 | 0.977 | 0.945 – 1.000 |
| **Aβ42/t-tau** | Lumipulse | 0.933 | 0.870 – 0.995 | 0.950 | 0.894 – 1.000 |
|  | INNOTEST | 0.959 | 0.910 – 1.000 | 0.978 | 0.946 – 1.000 |
| **Aβ42/p-tau181** | Lumipulse | 0.966 | 0.918 – 1.000 | 0.975 | 0.940 – 1.000 |
|  | INNOTEST | 0.955 | 0.910 – 1.000 | 0.977 | 0.945 – 1.000 |

Supplementary figure 1: Interference of spiked recombinant A40 with measurement of A42 by the three platforms.


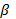

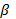

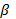

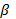


| A | = -.186 (-.732, .361) 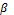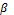 rho = -.055, *P* = .908 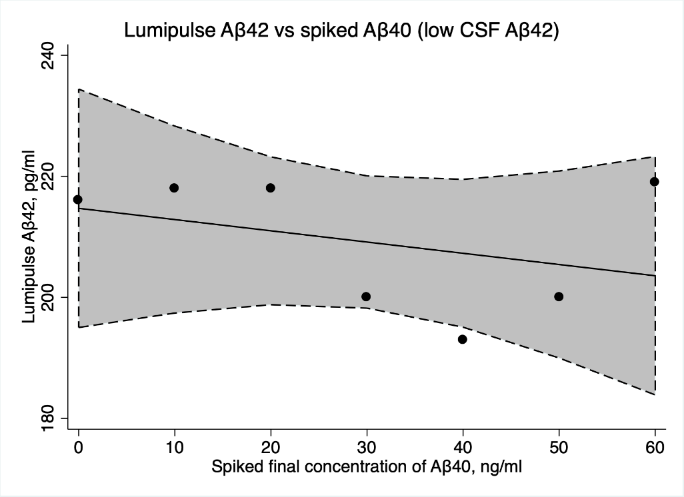 | B | = -.624 (-1.055, -.193) 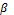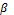 rho = -0.893, *P* = .007 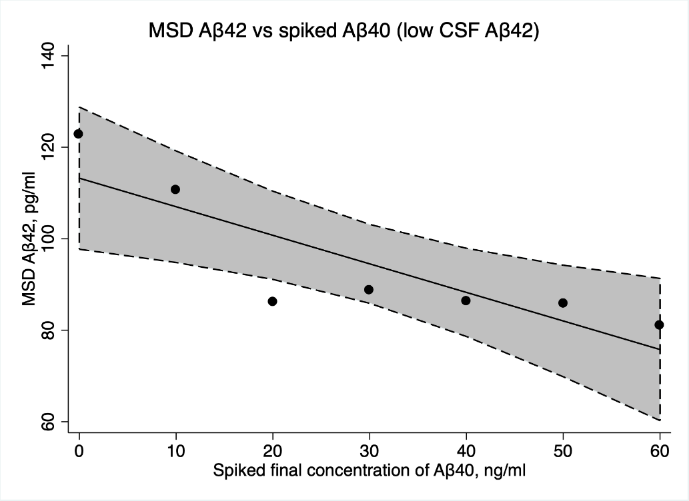 | C | = 1.665 (-2.1634, -1.166), rho = 0.964, *P* = .0005 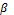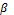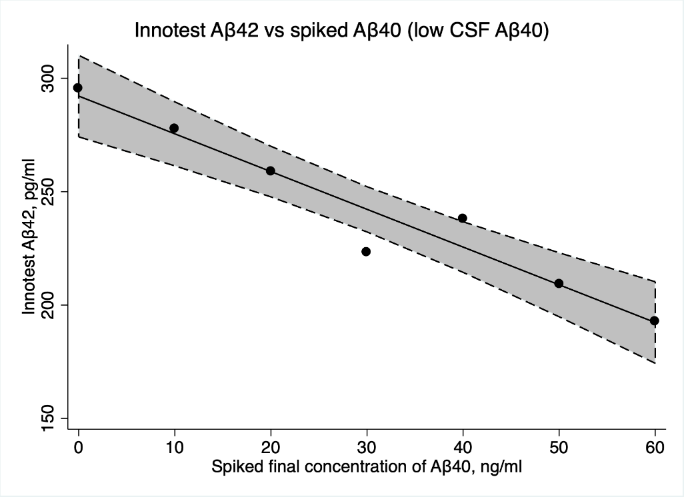 |
| --- | --- | --- | --- | --- | --- |
| D | = -.196 (-7.444, 7.051) 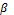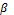 rho = 0.107, *P* = .819 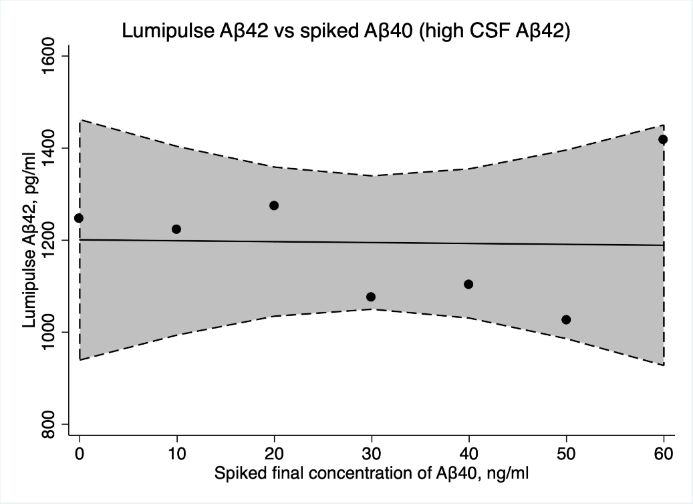 | E | = -5.121 (-8.084, -2.159) 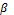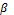 rho = -0.786, *P* = .036 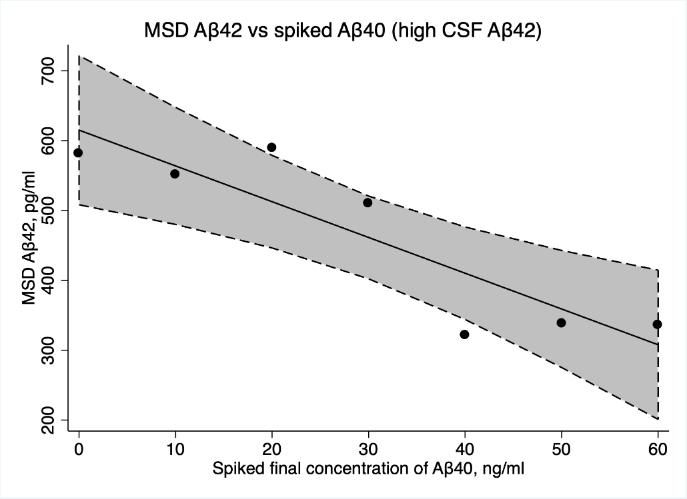 | F | = 5.246 (-7.878, -2.613), 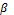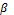 rho = -0.964, *P* = .0005 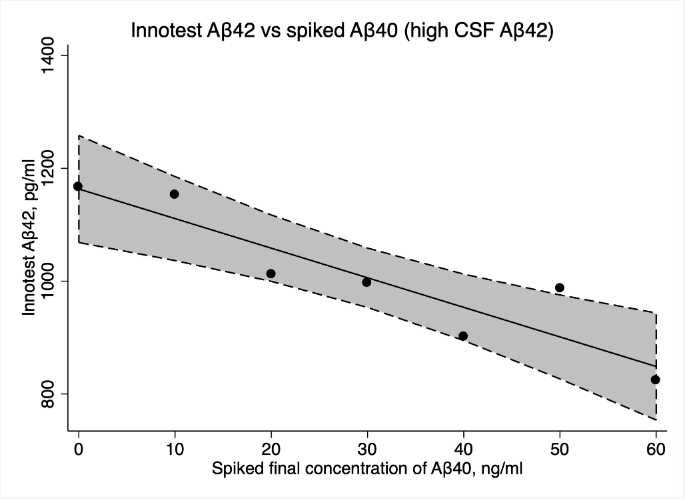 |
| G | = -.086 (-.339, .167) 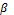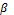 rho = -.055, *P* = .908 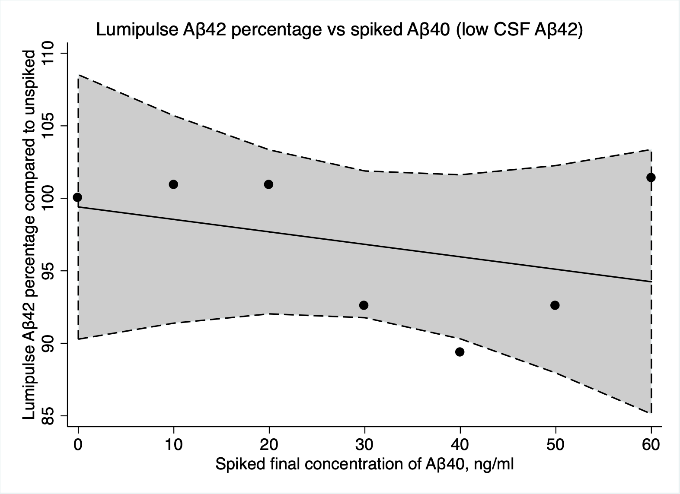 | H | = -.508 (-.859, -.157) 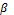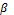 rho = -.893, *P* = .007 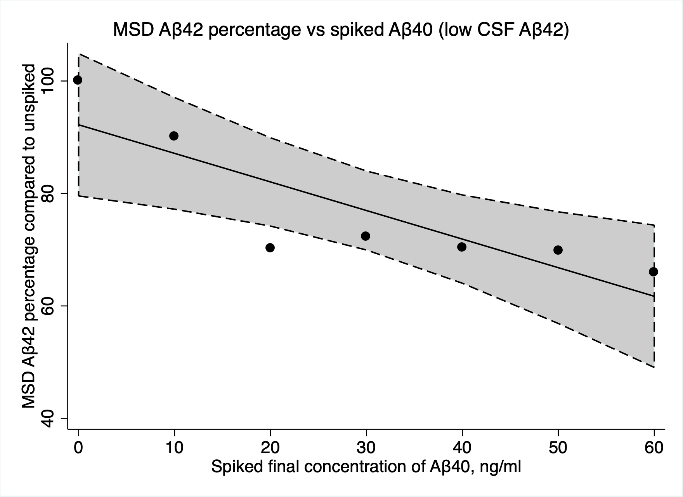 | I | = -.563 (-.732, -.394) 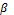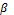 rho = -.964, *P* = .0005 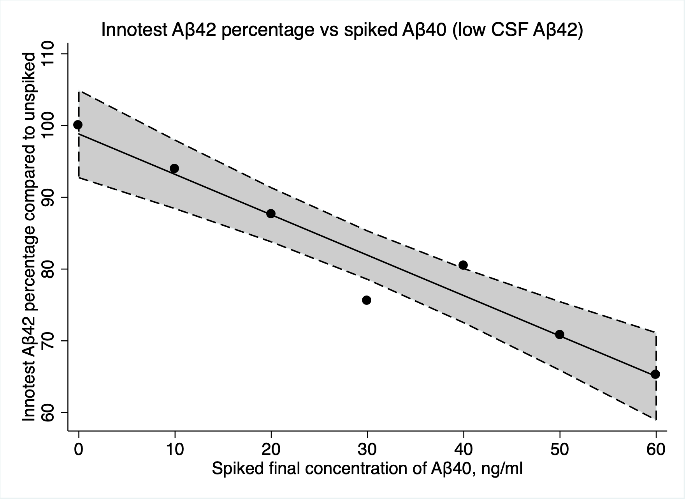 |
| J | = -.016 (-.597, .565) 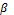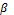 rho = -.107, *P* = .819 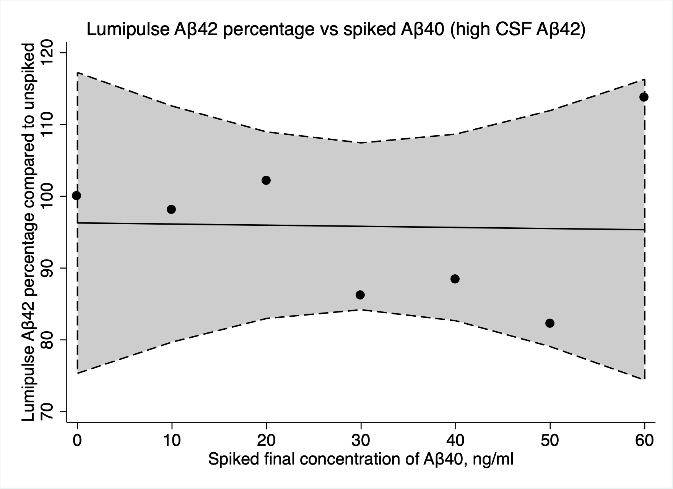 | K | = -.880 (-1.389, -.371) 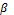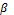 rho = -.786, *P* = .036 | L | = -.450 (-.675, -.224)  rho = -.964, *P* = .0005 |

The top row (A, B, C) shows the results of spiking a low CSF A42 sample, and the second row (D, E, F) a high CSF A42 sample, in terms of absolute A42 concentrations. The lower two rows (G, H, I and J, K, L) show the corresponding results in terms of percentage changes in A42 concentrations relative to the unspiked sample. Each point is the average of duplicate measurements. The solid line shows the linear correlation between the measured CSF A42 value and the known spiked concentration of A40, and the grey area its 95% confidence interval. The slope of the line (95% confidence interval), the Spearman correlation coefficient rho and its P value are shown below each graph.

Supplementary figure 2: Use of individual biomarkers vs ratios in separating amyloid PET negative (n=50) and positive (n=13) individuals.

| A |  | B |  | C |  |
| --- | --- | --- | --- | --- | --- |
| D |  | E |  | F |  |

Blue lines indicate the ratio cut-point (Aβ42/Aβ40 ratio – Panel A and B; Aβ42/t-tau ratio – Panel C and D; Aβ42/p-tau181 ratio – Panel E and F). Dashed lines denote cut-points for the individual biomarkers that performed better than chance. Cut-points were determined by the Youden J index. The ^18^F-florbetapir amyloid PET SUVR cut-point was 0.6104.

| A |  | B |  | C |  |
| --- | --- | --- | --- | --- | --- |
| D |  | E |  | F |  |

Supplementary figure 3: Scatter plots of predictions of models incorporating age, sex, APOE ɛ4 carrier status and CSF biomarker ratios (y axis) against SUVR (x axis), n=62

Dashed horizontal lines show the Youden’s index cut-points for the CSF ratios, above which an individual was classified as CSF–positive; dashed vertical lines show the ^18^F-florbetapir amyloid PET SUVR cut-point, to the right of which an individual was classified as amyloid PET–positive.
